# Comparison of Nonspherical Polyvinyl Alcohol Particles and Microspheres for Prostatic Arterial Embolization in Patients with Benign Prostatic Hyperplasia

**DOI:** 10.1155/2017/8732351

**Published:** 2017-06-22

**Authors:** Jin Ho Hwang, Sang Woo Park, Il Soo Chang, Sung Il Jung, Hae Jeong Jeon, Yong Soo Lho, Hyeong Gon Kim, Sung Hyun Paick, Hyoung Keun Park

**Affiliations:** ^1^Department of Radiology, Konkuk University Medical Center, Konkuk University School of Medicine, Seoul, Republic of Korea; ^2^Soo Radiology Clinic, Guri-si, Gyeonggi-do, Republic of Korea; ^3^Department of Urology, Dongin Hospital, Gangneung-si, Gangwon-do, Republic of Korea; ^4^Department of Urology, Konkuk University Medical Center, Konkuk University School of Medicine, Seoul, Republic of Korea

## Abstract

**Purpose:**

To report early results following prostatic artery embolization (PAE) and compare outcomes between nonspherical polyvinyl alcohol (PVA) particles and microspheres to treat lower urinary tract symptoms (LUTS) due to benign prostatic hyperplasia (BPH).

**Methods:**

PAE was performed in nine patients (mean age: 78.1 years) with symptomatic BPH. Embolization was performed using nonspherical PVA particles (250–355 *μ*m) in four patients and microspheres (300–500 *μ*m) in five patients.

**Results:**

PAE was technically successful in all nine patients (100%). During a mean follow-up of 10.1 months, improvements in mean International Prostate Symptom Score (IPSS), Quality of Life (QoL), prostatic volume (total volume and transition zone), and peak urinary flow (*Q*_max_) were 9.8 points, 2.3 points, 28.1 mL, 17.8 mL, and 4.5 mL/s, respectively. Clinical success was obtained in seven of nine patients (78%). Patients in the microsphere group showed greater improvement in IPSS, QoL, prostatic volume, and *Q*_max_ compared to patients in the nonspherical PVA particle group. However, significant difference was noted only in the prostatic volume.

**Conclusion:**

PAE is a feasible, effective, and safe treatment option for BPH with LUTS. Use of microspheres showed greater prostatic volume reduction compared to nonspherical PVA particles.

## 1. Introduction

Benign prostatic hyperplasia (BPH) is prevalent in men over 50 years of age [1, 2] and frequently causes lower urinary tract symptoms (LUTS) consisting of an incomplete emptying sensation, hesitancy, decreased urinary stream, urinary frequency, urgency, and nocturia [3, 4].

The gold-standard treatment for BPH is surgery via transurethral prostatic resection (TURP) [5]. However, surgical treatment, even laser enucleation or photovaporization, is associated with complications such as irritating urinary symptoms, retrograde ejaculation, impotence, and hemorrhage [6, 7]. Therefore, treatment of BPH-related symptoms is often initiated pharmacologically using *α*-blockers and 5-*α* reductase inhibitors [5]. Even though long-term pharmacotherapy can be effective, it is also associated with adverse effects such as dizziness, orthostatic hypotension, headache, erectile disorders, impotence, and decreased libido. Moreover, long periods of medication are hard to maintain due to poor compliance, high costs, and drug interactions [8].

For these reasons, the need to develop new, less invasive treatment modalities for BPH is increasing. Since DeMeritt et al. [9] reported a case of the reduction of prostatic volume after prostatic artery embolization (PAE) for the treatment of BPH with bleeding, experimental animal studies [10, 11] in dogs and pigs demonstrated that PAE can lead to prostate gland volume reduction without sexual dysfunction. Furthermore, a number of short-, intermediate-, and long-term studies in humans have shown that PAE is a safe, effective, and feasible treatment for the relief of LUTS associated with BPH [8, 12–17].

To date, research concerning the results of PAE using two different types of embolic agents has been performed: polyvinyl alcohol (PVA) particles and microspheres of various sizes. Nevertheless, the embolic agent of choice for PAE is yet to be determined.

The purpose of the present study was to investigate the feasibility, effectiveness, and safety of PAE in patients with LUTS due to BPH. We also aimed to compare the efficacy of 250–355 *μ*m nonspherical PVA particles and 300–500 *μ*m microspheres.

## 2. Methods

### 2.1. Study Population

The study was approved by the hospital's ethics committee, and all patients gave their informed consent to undergo PAE as an alternative treatment. All patients were evaluated by urologists and provided opportunities to receive other therapeutic options, including TURP or minimally invasive surgery.

From April 2011 to March 2014, a total of nine patients (age range: 50–91 years; mean: 78.1 years) presented with LUTS related to BPH and underwent PAE.

The inclusion criteria were age > 50 years, LUTS due to BPH refractory to medical treatment for >6 months, prostate volume > 30 mL, International Prostate Symptom Score (IPSS) ≥ 18 points or Quality of Life (QoL) score ≥ 3 points, peak urinary flow (*Q*_max_) ≤ 12 mL/s, or acute urinary retention.

The exclusion criteria were presence of prostatic malignancy evaluated by digital rectal exam, prostate-specific antigen (PSA), and transrectal ultrasound (TRUS). We also excluded patients with chronic renal failure, active urinary tract infection, and neurogenic bladder. In patients with a PSA level > 4.0 ng/mL, we performed TRUS-guided prostatic biopsy to exclude prostatic malignancy.

Patient selection was accomplished using a multidisciplinary approach in collaboration with urologists and interventional radiologists. Five patients were unsuitable for surgical treatment because of advanced age (>80 years old), one patient had cardiac disease (hypertrophic cardiomyopathy), and the remaining three patients refused surgical treatment. Five patients experienced acute urinary retention and received bladder catheters (four cystostomy catheters and one Foley catheter) the day before PAE.

Before the procedure, all patients were evaluated by clinical observation with measurement of IPSS and QoL scores, uroflowmetry (*Q*_max_ and postvoid residual urine), PSA levels, and TRUS examination to calculate prostate volume (total and transition zone volume).

Baseline data were obtained before PAE and the result of treatment was measured in one patient at 1 month, two at 3 months, two at 6 months, two at 12 months, and two at 24 months after PAE. The baseline data are summarized in Table 1.

US examination was performed using an HDI 5000 US scanner (Philips/ATL, Bothell, WA, USA) with an intracavitary probe (7 MHz), and the volume of the prostate gland was assessed using the following ellipsoid formula: *π*/6 × three-directional prostatic diameters (transverse × anteroposterior × cephalocaudal). Total volume and transition zone volume were calculated. One experienced genitourinary radiologist performed all TRUS exams.

### 2.2. Embolization Technique

All patients stopped taking their BPH medication 1 week before PAE. Patients received antibiotics (ciprofloxacin 400 mg, Shin Poong Pharm, Seoul, Korea) in a single intravenous dose before the procedure, followed by oral medication (ciprofloxacin 500 mg, Je IL Pharm, Daegu, Korea, twice daily) for 7 days after PAE. All patients received analgesic medication (pethidine 25 mg, Je IL Pharm, Daegu, Korea) immediately before the procedure. Patients were given nonsteroidal anti-inflammatory medication (airtal 100 mg, Daewoong Pharm, Seoul, Korea, twice daily) and acid-suppressing drugs (omeprazole 20 mg, Yuhan Co., Seoul, Korea) for 7 days following PAE.

The procedure was performed by one experienced interventional radiologist in an angiography suite (Axiom Artis; Siemens, Erlangen, Germany). Embolization was performed under local anesthesia with a unilateral approach, usually via the right femoral arterial access. First, pelvic angiography was performed to evaluate the internal iliac and prostatic arteries. Then, selective bilateral internal iliac arteriograms were obtained using a 5-F angiographic catheter (Yashiro catheter; Terumo, Tokyo, Japan) in the anterior-posterior (AP) and ipsilateral 35° oblique view with nonionic contrast medium (Iomeron 350; Bracco, Milan, Italy).

We performed prostatic arterial catheterization using a 2.0-F microcatheter (Progreat; Terumo, Tokyo, Japan) and a 0.014-inch guidewire (Transcend; Boston Scientific, Natick, USA), and prostatic arteriography was performed by manual injection in the AP projection. Embolization was performed using 250–355 *μ*m nonspherical PVA particles (Contour; Boston Scientific, Natick, USA) in four patients (eight prostatic arteries) and 300–500 *μ*m microspheres (Contour SE; Boston Scientific, Natick, USA) in five patients (nine prostatic arteries). The embolic material used in each patient was randomly selected. Contour and Contour SE were diluted in 20 mL of normal saline and 30 mL of contrast medium in a 2 : 3 solution. The particles were slowly injected through a 1 mL syringe under fluoroscopic control until we reached an end point of near stasis of contrast agent without reflux of embolic agent, avoiding nontarget embolization of undesired arteries.

We declared the procedure successful when superselection of the prostatic artery with microcatheter and injection of embolic materials into prostatic artery was achieved. Clinical success was defined as improvement of LUTS (IPSS reduction of at least 25% of the total score and lower than 18 points) and QoL (QoL reduction of at least 1 point or below 3 points) on follow-up or removal of indwelling bladder catheters in patients with urinary retention before PAE.

Postembolization symptoms and complications were assessed according to the quality improvement guidelines for percutaneous transcatheter embolization [18]. Complications were considered as minor if they could be addressed by outpatient medical treatment and major if they resulted in prolonged hospitalization, readmission, or additional surgery. We measured the procedure time from femoral arterial puncture to catheter removal after PAE.

### 2.3. Statistical Analysis

Statistical analysis was performed using SPSS, version 23 (IBM Inc., Chicago, IL, USA). We used the Wilcoxon signed-rank test to compare baseline and outcome variables. To compare data between the microsphere and nonspherical PVA particle groups, the Mann–Whitney *U* test was used. A *P* value < 0.05 was considered statistically significant.

## 3. Results

**Figure 1:**
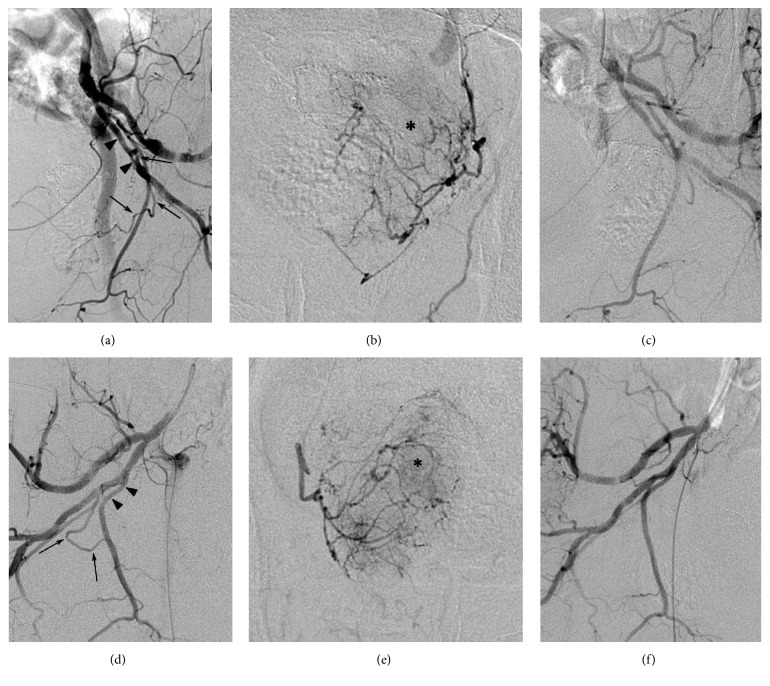
Arteriographic images of a 76-year-old patient having lower urinary tract symptoms associated with benign prostatic hyperplasia who underwent bilateral prostatic artery embolization. Arteriograms obtained on both pelvic sides before (a, b, d, and e) and after (c and f) embolization under AP (b and e) and ipsilateral oblique views (a, c, d, and f) showing the bilateral prostatic arteries (arrows). The left prostatic artery originated from the inferior gluteal artery (arrowheads, a), and the right one originated from the internal pudendal artery (arrowheads, d). In the parenchymal phase of prostatic arteriography, prostate glands (asterisks, b and e) are opacified by bilateral prostatic arteries. After embolization using 300–500 *μ*m microspheres, the bilateral prostatic arteries were successfully embolized (c and f).

PAE was technically successful in all nine patients (100%). We performed bilateral PAE in eight patients (89%) and unilateral PAE in one patient because of unilateral agenesis or atherosclerotic occlusion of the prostatic artery. The origins of prostatic arteries were as follows: internal pudendal artery (*n* = 11, 64.7%), gluteal-pudendal trunk (*n* = 3, 17.6%), obturator artery (*n* = 2, 11.8%), and inferior gluteal artery (*n* = 1, 5.9%) (Figure 1).

PAE procedure time ranged from 20 to 202 min (mean, 79 min) and the fluoroscopy time was between 8 and 84 min (mean, 24 min). Mean follow-up was 10.1 months (range, 1–24 months).

Seven patients were discharged the day after the procedure, and the remaining two patients were discharged 2 and 3 d after PAE. The reasons for delayed discharge were reinsertion of a cystostomy catheter caused by spontaneous removal of the catheter in one patient, and correction of electrolyte imbalance associated with prolonged diarrhea started before the procedure in the other patient. Mean hospitalization was 2.8 d (range, 2–6 days).

At the end point of follow-up after PAE, IPSS improved from 24.6 ± 9.7 to 14.8 ± 9.4 points (mean improvement of 9.8; *P* = 0.011) and QoL score improved from 4.9 ± 1.1 to 2.6 ± 1.3 (mean improvement of 2.3; *P* = 0.011).

**Figure 2:**
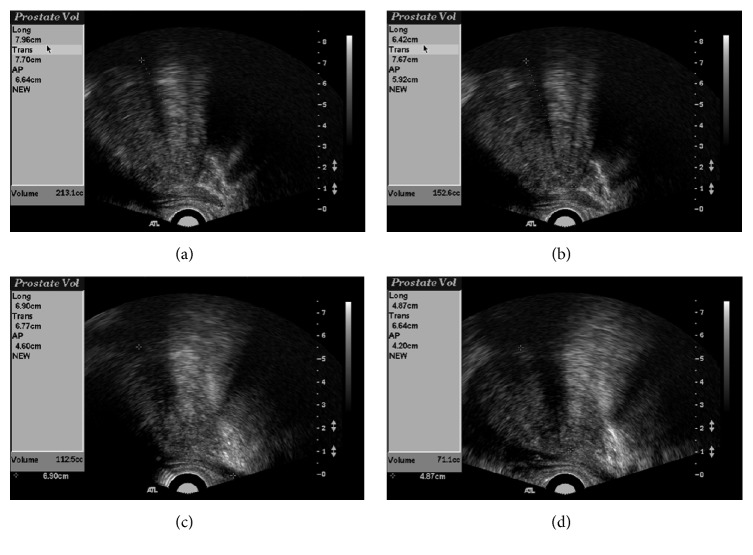
Transrectal ultrasound (US) images before embolization and at follow-up after prostatic artery embolization (PAE) (the same case as shown in Figure 1). Total volume (a and c) and transition zone volume (b and d) were obtained. US images obtained before PAE (a and b) and 24 months after PAE (c and d) showing marked reduction in prostatic volume.

The total prostatic volume in nine patients decreased from 89.4 ± 59.3 to 61.3 ± 31.2 mL (mean decrease of 28.1 mL; *P* = 0.008), and the prostatic volume of the transition zone decreased from 59.5 ± 43.7 to 41.7 ± 25.1 mL (mean decrease of 17.8 mL; *P* = 0.008) at the last follow-up (Figure 2). *Q*_max_ improved from 5.2 ± 4.7 to 9.8 ± 6.2 mL/s (mean increase of 4.5 mL/s; *P* = 0.011). The changes in study values before and after PAE are shown in Table 2.

Out of five patients who received vesical catheters due to acute urinary retention before PAE, indwelling catheters were removed within 2 months after PAE in three patients who were able to urinate successfully. In two patients (91 and 86 years old), removal of the catheter was impossible due to failure of voluntary urination. These two patients had persistent severe LUTS after PAE (IPSS and QoL above 18 and 4) despite a reduction in total prostate volume of 15.4% and 29.7%. Clinical success was obtained in seven of nine patients (78%).

In one patient who underwent unilateral PAE, improvement of IPSS (16 points), QoL (4 points), and *Q*_max_ (7 mL/s) and reduction of total (26.7%) and transitional (28.9%) prostatic volume were obtained. He had his cystostomy catheter removed 3 weeks after PAE.

The results of clinical outcomes between groups using microspheres or nonspherical PVA particles are provided in Table 3. Patients in the microsphere group had a greater decrease in IPSS (11.6 ± 6.2 versus 7.5 ± 6.5 points), QoL (2.6 ± 1.1 versus 2.0 ± 1.6 points), and total (29.5 ± 11.5 versus 20.9 ± 11.5%) and transition zone (30.5 ± 15.7 versus 17.1 ± 7.3%) prostatic volume and a greater increase in *Q*_max_ (5.4 ± 2.7 versus 3.4 ± 3.8 mL/s) compared to patients in the nonspherical PVA particle group. Except for prostatic volume (total and transition zone), no significant differences in IPSS, QoL, and *Q*_max_ were noted between the two groups (Table 3). There was one patient in each group in whom clinical success could not be obtained after PAE.

No major complications were noted in this study, but minor complications were seen in one patient who experienced mild penile pain on the day following PAE. The penile pain disappeared spontaneously the day after symptom occurred without the need for further treatment.

## 4. Discussion

Since DeMeritt et al. [9] indicated prostatic volume reduction and improvement of LUTS after management for prostatic bleeding using PAE, experimental animal studies in dogs and pigs have shown that PAE is an effective and safe procedure for reducing prostatic volume [10, 11]. Sun et al. [11] suggested a mechanism for PAE consisting of prostate cell death and necrosis induced by ischemia and decreased levels of free plasma testosterone (static pathologic component) and decreased numbers of *α*_1_-adrenergic receptors associated with prostatic neuromuscular tone (dynamic pathologic component).

The first case of PAE specifically for the treatment of BPH was reported by Carnevale et al. [12] in two patients who experienced volume reductions of 47.8% and 27.8%. Several subsequent studies showed variable degrees of mean prostatic volume reduction from 18% to 32% [8, 14, 19, 20]. In the present study, there was a significant mean reduction of 28.1 mL (31.4%) in total prostatic volume and 17.8 mL (29.9%) in transition zone prostatic volume after PAE. With respect to *Q*_max_, the mean increase was 4.5 mL/s in the present study, and other studies reported improvements in *Q*_max_ of 3.85–6.6 mL/s [14, 21, 22]. In terms of subjective clinical parameters (IPSS and QoL score), there were improvements of 9.8 and 2.3 points, respectively. These results were comparable to those of previous studies (2.8 to 19 in IPSS and 0.4 to 2.5 in QoL) [14, 16, 17, 21].

The optimal type (spherical or nonspherical) or size of embolic agents has not yet been determined. In animal experimental studies [10, 11] demonstrating the efficacy and safety of PAE, 500–700 *μ*m microspheres and 250–355 *μ*m PVA particles were used. However, smaller (150–250 *μ*m) PVA particles were used in the first report of PAE in a human patient [9]. Subsequent studies [8, 12, 15–17, 23, 24] have demonstrated the use of variable sizes (from less than 100 *μ*m up to 500 *μ*m) of PVA particles or microspheres. Although Jeon et al. [10] suggested that smaller PVA particles may elicit better effects in PAE with further penetration into the periphery, there are concerns about nontarget embolization because of anastomoses between the prostatic arteries and neighboring arteries [25]. Furthermore, Brook et al. [26] took another view that larger embolic particles may induce greater prostatic volume reductions and better results after PAE in a canine model. They considered 500–700 *μ*m microspheres as reasonable particle sizes for PAE in canine BPH. In the present study, we used 250–355 *μ*m nonspherical PVA particles and 300–500 *μ*m microspheres as embolic agents, and reductions in prostatic volume (31.4%) and improvements in *Q*_max_ (4.5 mL/s) were achieved. The result was comparable to the reports [16, 17, 24] using <200 *μ*m particles as an embolic agent.

A randomized trial [24] that evaluated different sizes of PVA particles and their results was reported. However, as far as we know, there have been no reports comparing microspheres and nonspherical PVA particles in PAE. As Table 3 shows, in terms of the clinical outcomes measured by subjective (IPSS and QoL) and objective values (prostatic volume and *Q*_max_), the microsphere group had better outcomes compared to the nonspherical PVA particle group in descriptive statistics. Regarding prostate gland volume reduction, statistical significance was achieved in the total prostate and the transition zone. It is believed that nonspherical PVA particles have more chance to perform proximal embolization than microspheres, which may reduce the possibility of clinical success [27]. We suspect that the better results in the microsphere group may be attributable to the reasons mentioned above. However, in our study, the statistical analysis failed to reveal significant differences in values including IPSS, QoL, and *Q*_max_ between the two groups, most likely due to the limited sample size.

There is no direct correlation between prostate volume reduction and the clinical symptom improvement. According to a previous report [15] of 52 patients in whom clinical success could not be obtained, 23 patients experienced clinical failure despite significant (more than 15%) prostate volume reductions after PAE. We achieved a clinical success of 78% (seven of nine patients). Two patients who were experiencing clinical failure experienced significant volume reductions of 15.4% and 29.7%. In contrast, one patient experienced clinical success in spite of an insignificant volume reduction of 8%.

Unilateral prostatic arterial embolization may cause sufficient prostatic ischemia to improve symptoms. Bilhim et al. [22] reported better clinical outcomes in bilateral PAE than unilateral PAE (75% versus 50%). However, they demonstrated reasonable efficacy with unilateral PAE due to the existence of anastomoses between the bilateral prostatic arteries [25]. Carnevale et al. [12] reported one patient who underwent unilateral PAE and experienced a prostate volume reduction of 27.8%. In the present study, one patient underwent unilateral PAE and experienced a decrease in total prostatic volume reduction (26.7%) and improvement in *Q*_max_ (7 mL/s), IPSS (16 points), and QoL score (4 points). These results were comparable to those of bilateral PAE, which showed a mean volume reduction of 31.4% and mean improvements of *Q*_max_ of 3.4 mL/s, IPSS of 9 points, and QoL score of 2.1 points.

Prostatic arteries have small diameters of less than 2 mm, and their origin is highly variable. Thorough understanding of prostatic arterial anatomy is important to avoid embolization failure and nontarget embolization of the rectum, bladder, and penis [14, 25, 28, 29]. Bilhim et al. [25] reported the origin of prostatic arteries as follows: internal pudendal artery (56%), common gluteal-pudendal trunk (28%), obturator artery (12%), and inferior gluteal artery (4%). In this study, the most common prostatic artery origin was the internal pudendal artery (64.7%). The next most frequent artery origins were the gluteal-pudendal trunk (17.6%), obturator artery (11.8%), and inferior gluteal artery (5.9%). These frequencies of prostatic arterial origin were similar to those of the results of the study described above.

Open prostatectomy is the procedure of choice for BPH larger than 80 cm^3^ [30]. Recently, several reports have suggested that PAE for prostate volume greater than 80 cm^3^ is safe and effective [17, 27, 31, 32]. These reports reflect the growing interest in PAE, especially in patients who are not candidates for open surgery, TURP, or minimally invasive surgery. In the present study, there were five patients with a prostate volume larger than 80 cm^3^ (range, 87.5–213.1 mL; mean, 127.5 mL). The outcomes after PAE were as follows: IPSS (mean improvement of 11.6), QoL (mean improvement of 2.6), *Q*_max_ (mean increase of 5.2 mL/s), and total prostatic volume (mean decrease of 43.9 mL, 31.6%), and these results are consistent with those of previously reported studies.

There are some limitations to the present study. First, we included a small number of patients and the end point of follow-up was not the same for each patient. Due to the insufficient numbers of patients in each group, statistical significant was not achieved with respect to several values. Larger comparative studies concerning the type or size of embolic agents for PAE will help physicians to choose the most appropriate embolic material. Second, the PSA levels after PAE were not checked appropriately. PSA level reflects prostatic inflammation and the degree of ischemia, and we missed the opportunity to analyze the change of PSA level after PAE.

In conclusion, PAE is feasible, effective, and safe for the treatment of BPH with LUTS. Regarding the total and transition zone volumes of the prostate gland, the use of microspheres (300–500 *μ*m) is associated with greater reductions than nonspherical PVA particles (250–355 *μ*m).

## Figures and Tables

**Table 1:** Baseline characteristics of the nine patients.

Characteristics	Value (mean ± SD)	Range
Age (y)	78.1 ± 12.3	50–91
IPSS (point)	24.6 ± 9.7	8–35
QoL score	4.9 ± 1.1	3–6
PV (total) (mL)	89.4 ± 59.3	35.3–213.1
PV (transition zone) (mL)	59.5 ± 43.7	17.8–152.6
*Q*_max_ (mL/s)	5.2 ± 4.7	0–11.9
PSA (ng/mL)	9.5 ± 11.1	0.5–28.9

IPSS, International Prostate Symptom Score; QoL, Quality of Life; PV, volume of prostate; *Q*_max_, peak urinary flow; PSA, prostate-specific antigen.

**Table 2:** Changes in study values before and after prostatic artery embolization.

Variables	Value		Mean difference	*P* value
	Before	After		
IPSS (point)	24.6 ± 9.7	14.7 ± 9.4	−9.8 ± 6.3	0.011
QoL score	4.9 ± 1.1	2.6 ± 1.3	−2.3 ± 1.3	0.011
PV (total) (mL)	89.4 ± 59.3	61.3 ± 31.2	−28.1 ± 30.1	0.008
PV (transition zone) (mL)	59.5 ± 43.7	41.7 ± 25.1	−17.7 ± 24.5	0.008
*Q*_max_ (mL/s)	5.2 ± 4.7	9.8 ± 6.2	4.5 ± 3.2	0.011

IPSS, International Prostate Symptom Score; QoL, Quality of Life; PV, volume of prostate; *Q*_max_, peak urinary flow.

**Table 3:** Comparison of clinical responses after prostatic artery embolization between the two embolic agent groups.

Variables	Microsphere (*n* = 5)		Nonspherical PVA (*n* = 4)		*P* value
	Before	After	Before	After	
IPSS	27.2 ± 9.5	15.6 ± 8.0	21.3 ± 10.1	13.8 ± 12.1	0.319
QoL score	5.0 ± 1.0	2.4 ± 1.1	4.8 ± 1.3	2.8 ± 1.7	0.530
PV (total) (mL)	117.3 ± 66.0	76.9 ± 32.3	54.6 ± 25.5	41.9 ± 17.0	0.050
PV (transition zone) (mL)	82.0 ± 46.0	54.5 ± 24.7	31.3 ± 19.4	25.8 ± 16.0	0.014
*Q*_max_ (mL/sec)	4.4 ± 4.9	9.8 ± 7.4	6.3 ± 4.8	9.7 ± 5.5	0.462

IPSS, International Prostate Symptom Score; QoL, Quality of Life; PV, volume of prostate; *Q*_max_, peak urinary flow.
